# Candidate Bioinks for Extrusion 3D Bioprinting—A Systematic Review of the Literature

**DOI:** 10.3389/fbioe.2021.616753

**Published:** 2021-10-13

**Authors:** Sam P. Tarassoli, Zita M. Jessop, Thomas Jovic, Karl Hawkins, Iain S. Whitaker

**Affiliations:** ^1^Reconstructive Surgery & Regenerative Medicine Research Group (ReconRegen), Swansea University Medical School, Institute of Life Sciences, Swansea, United Kingdom; ^2^The Welsh Centre for Burns & Plastic Surgery, Morriston Hospital, Swansea, United Kingdom; ^3^Centre for NanoHealth, Swansea University Medical School, Institute of Life Sciences, Swansea, United Kingdom

**Keywords:** bioprinting, bioink, extrusion, systematic review, regenerative medicine

## Abstract

**Purpose:** Bioprinting is becoming an increasingly popular platform technology for engineering a variety of tissue types. Our aim was to identify biomaterials that have been found to be suitable for extrusion 3D bioprinting, outline their biomechanical properties and biocompatibility towards their application for bioprinting specific tissue types. This systematic review provides an in-depth overview of current biomaterials suitable for extrusion to aid bioink selection for specific research purposes and facilitate design of novel tailored bioinks.

**Methods:** A systematic search was performed on EMBASE, PubMed, Scopus and Web of Science databases according to the PRISMA guidelines. References of relevant articles, between December 2006 to January 2018, on candidate bioinks used in extrusion 3D bioprinting were reviewed by two independent investigators against standardised inclusion and exclusion criteria. Data was extracted on bioprinter brand and model, printing technique and specifications (speed and resolution), bioink material and class of mechanical assessment, cell type, viability, and target tissue. Also noted were authors, study design (*in vitro*/*in vivo*), study duration and year of publication.

**Results:** A total of 9,720 studies were identified, 123 of which met inclusion criteria, consisting of a total of 58 reports using natural biomaterials, 26 using synthetic biomaterials and 39 using a combination of biomaterials as bioinks. Alginate (*n* = 50) and PCL (*n* = 33) were the most commonly used bioinks, followed by gelatin (*n* = 18) and methacrylated gelatin (GelMA) (*n* = 16). Pneumatic extrusion bioprinting techniques were the most common (*n* = 78), followed by piston (*n* = 28). The majority of studies focus on the target tissue, most commonly bone and cartilage, and investigate only one bioink rather than assessing a range to identify those with the most promising printability and biocompatibility characteristics. The Bioscaffolder (GeSiM, Germany), 3D Discovery (regenHU, Switzerland), and Bioplotter (EnvisionTEC, Germany) were the most commonly used commercial bioprinters (*n* = 35 in total), but groups most often opted to create their own in-house devices (*n* = 20). Many studies also failed to specify whether the mechanical data reflected pre-, during or post-printing, pre- or post-crosslinking and with or without cells.

**Conclusions:** Despite the continued increase in the variety of biocompatible synthetic materials available, there has been a shift change towards using natural rather than synthetic bioinks for extrusion bioprinting, dominated by alginate either alone or in combination with other biomaterials. On qualitative analysis, no link was demonstrated between the type of bioink or extrusion technique and the target tissue, indicating that bioprinting research is in its infancy with no established tissue specific bioinks or bioprinting techniques. Further research is needed on side-by-side characterisation of bioinks with standardisation of the type and timing of biomechanical assessment.

## Introduction

Bioprinting is defined as the precise and accurate deposition of biomaterials simultaneously with cells, within a predesigned space using a computer-aided printer (Langer and Tirrell, 2004; Mironov, 2005). The process was first described in 1988 (Klebe, 1988) as “cytoscribing” which involved depositing coloured inks in three-dimensions (3D), inspired by the two-dimensional (2D) paper printers that preceded them. Since then, biological materials have been used and increasingly complex printing methods developed which have made significant contributions to this evolving field (Lee et al., 2010; Benwood et al., 2021). These printable biomaterials, known as bioinks, can mimic the extracellular matrix (ECM) environment to support cell adhesion, differentiation and proliferation. Bioinks differ from other inks used in additive manufacturing (or 3D printing) in that they must serve both biological and mechanical functions. As such, they are printed at much lower temperatures, are typically derived naturally and have mild cross-linking conditions to preserve the cells and prevent unwanted degradation of the biomolecules (Malda et al., 2013).

Several bioprinting technologies have been explored, from inkjet and stereolithography to laser-assisted (3). Extrusion based techniques, also known as fused deposition modelling or bioplotting, are the most commonly used and involve extrusion of a viscous material containing cells, as a continuous filament through a nozzle using either a pneumatic, piston, or screw forces (Landers et al., 2002; Jakab et al., 2008). After printing, the constructs can be solidified (i.e., gelled) layer-by-layer either physically or chemically, which makes this technique slower than others such as laser assisted or ink jet based (Landers et al., 2002; Smith et al., 2004; Jakab et al., 2008). However, the cost effectiveness (many are made from modifying commercially available 3D printers), simplicity and high cell viability makes extrusion bioprinting a popular choice for most research institutions and is the basis of the first commercial 3D bioprinter (Organovo Novogen MMX Bioprinter^TM^) (Jürgen et al., 2016). The main limiting factors relate to achieving a high enough resolution to enable reproduction of native nano- and micro-architecture and maintaining post printing shape fidelity to enable bioprinting of complex macrostructures. The resolution itself will depend on the tissue type attempted to be recreated e.g., bundles and cell alignments for muscle bioprinting demanded a bioprinting resolution of <50 μm whereas some of the finest features of tissue microarchitectures required ~10 μm and mainly limited by the size of the individual cells themselves (Miri et al., 2019). These in turn depend on optimal material rheology and crosslinking ability (Jessop et al., 2017; Kyle et al., 2017).

The desirable characteristics of scaffolds for tissue engineering have been well-described (Hutmacher, 2000; Hollister, 2005; Al-Himdani et al., 2017), whereas those of bioinks are less well-defined (Kyle et al., 2017). The development and discovery of bioinks has had a staggered past with no natural research evolution (Tarassoli et al., 2018b). Initial studies used existing natural polymers (such as alginate or gelatin) for bioprinting rather than tailoring biomechanics to suit individual bioprinters (Boland et al., 2003; Jakab et al., 2004). These bioinks are still commonplace in modern bioprinting laboratory practise either alone or in combination with other biomaterials. More recent developments include smart polymers, consisting of synthetic materials with bioactive proteins to enable modification of biomechanical and biocompatibility properties, respectively (Galaev and Mattiasson, 1999). Their design allows for them to respond to specific stimuli (such as temperature, magnetism, ionic etc.) (Wei et al., 2017), but these are not necessarily optimised for extrusion bioprinting. Work has been carried out in the field of smart polymers in 3d bioprinting and cell therapy and is becoming an emerging avenue for further research (Huang et al., 2019).

**Figure 1:**
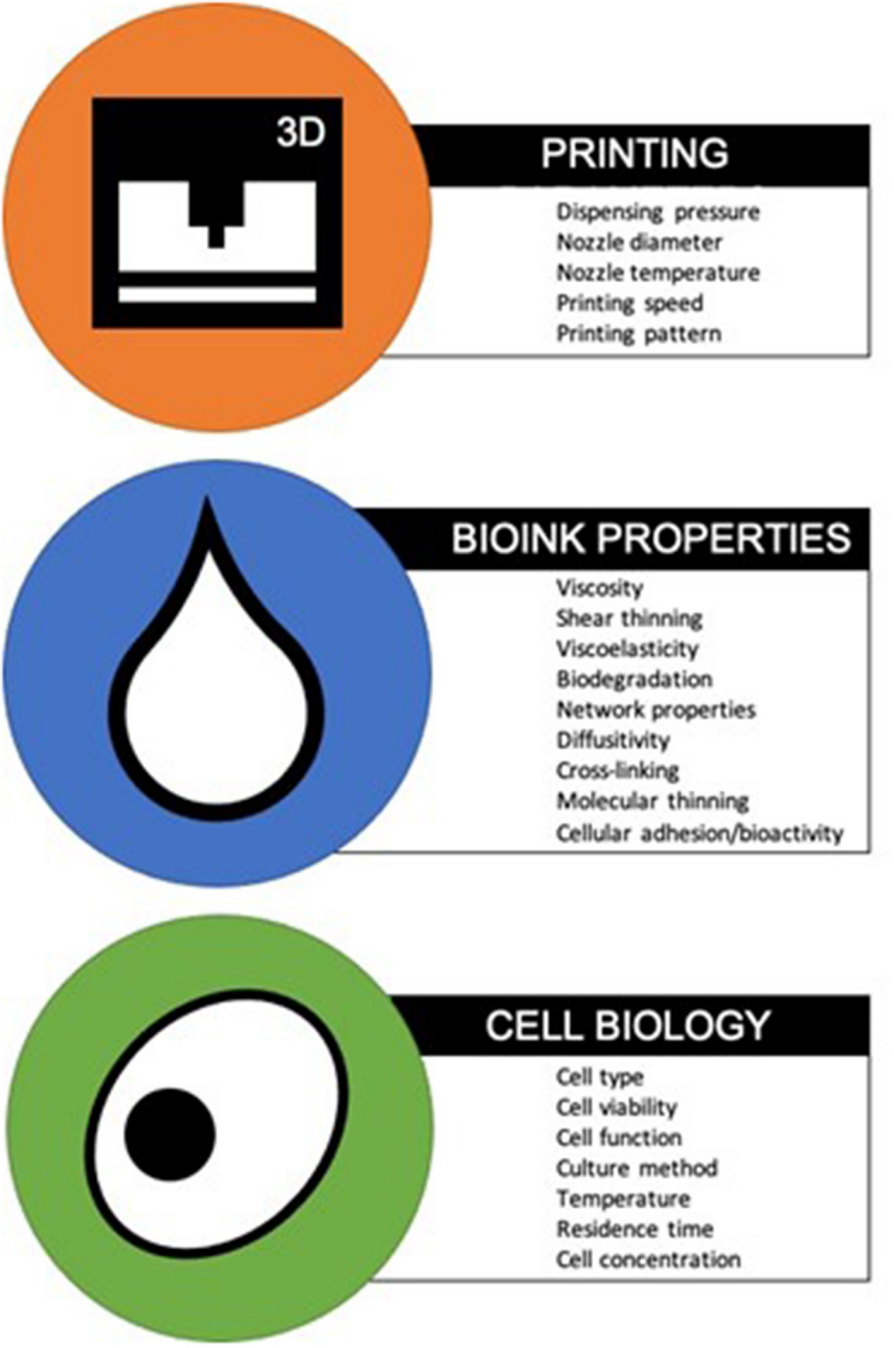
3D printer parameters, bioink properties, and cell characteristics for optimum bio-printability (Chang et al., 2008; Derby, 2012; Zhang and Cui, 2012; Kirchmajer et al., 2015).

Bioinks are classified as natural or synthetic. Natural bioinks differ to synthetic bioinks in their ability to mimic the native cellular microenvironment, offering support to growing cells and thereby increasing the likelihood of cell adhesion and secretion of matrix. On the other hand, synthetic bioinks are easier to tailor for efficient printability (Gopinathan and Noh, 2018). Many features of bioinks, such as biodegradability, ability to functionalise and sterilise, will naturally overlap with those of conventional scaffolds used for tissue engineering applications (Al-Himdani et al., 2017). However, the requirement for “printability” of bioinks introduces a new array of design criteria, encompassing parameters such as viscosity, viscoelasticity, cell supportive ability and gelation kinetics, which require further characterisation (Figure 1) (Ersumo et al., 2016; Kyle et al., 2017). Ideally, the bioink should possess the biomechanical properties to allow easy extrusion at low shear forces during printing, whilst supporting cell growth and maintaining shape fidelity post-printing (Jessop et al., 2017). Other considerations include biocompatibility with the chosen cell type, allowing not only cell survival but promotion of cellular proliferation, differentiation and extracellular matrix secretion to promote formation of the target tissue (Kyle et al., 2017). Taken together these features can be defined as the “bio-printability” properties of bioinks. Such customisable technology means that properties required of candidate bioinks may not be equally weighted; resolution may be more important for one type of application and cell supportive ability might be more sought after for another (Malda et al., 2013). In addition, biological variation in the behaviour of even identical cell lines means that outcomes can differ independently of the bioink, adding to the complexity of research in this field (Hutmacher, 2000; Ersumo et al., 2016).

Advanced bioinks are now being designed to significantly improve printability and biocompatibility (Kyle et al., 2017). These can be achieved through careful control of various physical, chemical and biological properties. Biomechanical properties that can be assessed include rheology (viscosity, shear-thinning, viscoelasticity, and thixotropy), gelation kinetics, crosslinking and network architecture (Murphy et al., 2013; Jia et al., 2014; Blaeser et al., 2016). Biocompatibility can also be altered through biofunctionalisation which can affect cell function (cytocompatibility, cell adhesion, migration, proliferation, and differentiation) as well as biodegradation of materials (Skardal et al., 2012; Levato et al., 2014; Muller et al., 2015; Daly et al., 2016). The tunability of these scaffolds also serve the purpose of helping protecting cells (from stresses) and allowing them to achieve the highest degree of viability (Sharma et al., 2020).

The aim of this systematic review was to identify the types of bioinks that have been used to date for extrusion-based 3D bioprinting and analyse their biomechanical characteristics, biocompatibility and bioprinting parameters so that these may aid in decision making when selecting suitable bioinks for individual purposes as well as the design of novel tailored bioinks.

## Materials and Methods

### Search Strategy

**Figure 2:**
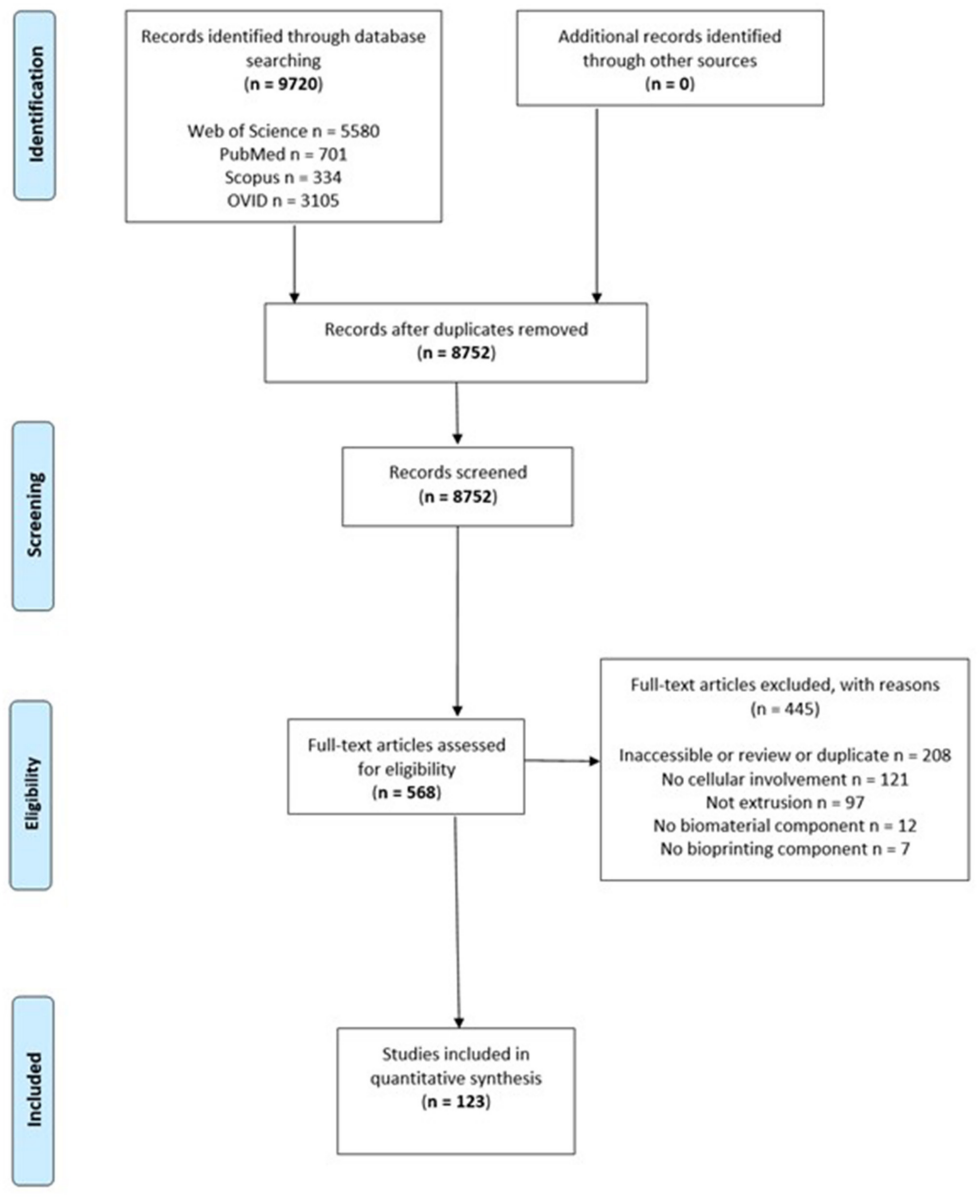
Preferred Reporting Items for Systematic Reviews and Meta-Analyses flow chart of included studies.

A systematic search for relevant articles was performed in accordance with the recommendations of the Preferred Reporting Items for Systematic Reviews and Meta-Analyses (PRISMA) guidelines (Figure 2) (Moher et al., 2015) to evaluate the types of biomaterials that have been used as bioinks for extrusion 3D bioprinting. Preclinical studies were identified through a systematic search across electronic databases Web of Science (Web of Science Core Collection, BIOSIS Citation index, KCI-Korean Journal Database, MEDLINE, SciELO Citation Index), PubMed, Scopus, OVID and Embase, from December 2006 to November 2017. Search terms included: “3d print” OR “3-dimensional print” OR “bioprint” OR “print” OR “additive manufacturing” OR “extrusion” OR “extrusion-based” OR “deposition” OR “pneumatic” OR “piston” OR “screw” AND (“bioink” OR “cell” OR “ink” OR “scaffold” OR “hydrogel”).

### Eligibility Criteria

The eligibility for including articles in this systematic review were as follows: (1) all studies included involved extrusion bioprinting; (2) either using animal or human cells; (3) both *in vitro* and *in vivo* studies; (4) English language articles only.

### Exclusion Criteria

Studies were excluded if they met the following criteria: (1) no bioprinting component; (2) involved non-extrusion based bioprinting techniques; (3) contained no biomaterial component; (4) contained no cellular component; (5) were not available for viewing. Review articles and commentaries were also excluded.

### Study Selection

**Figure 3:**
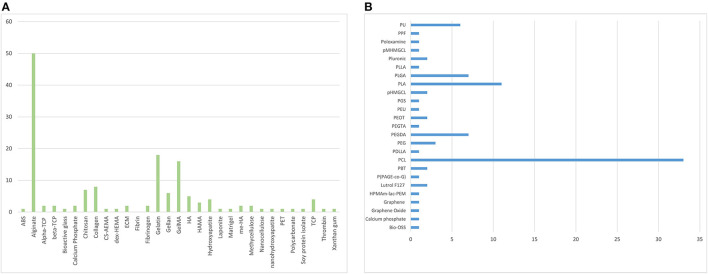
The individual distribution frequency of natural **(A)** and synthetic **(B)** bioinks used in the analysed literature.

Two reviewers (S.P.T. and Z.M.J.) independently reviewed the studies with differences resolved by the senior author (I.S.W.). The bibliographies of relevant articles were studied to identify further relevant publications (Figure 3). Titles were initially screened to exclude duplicates and further screened using the abstracts against inclusion and exclusion criteria. Finally, full text review of the remainder was performed to assess of eligibility.

### Data Extraction and Main Outcomes

Data was extracted from the selected studies using a standardised format (Microsoft Office Excel 2016). The initial tabulated data collection included; bioink composition, biomechanics (compressive, elastic and shear storage moduli, viscosity or as stated in Supplementary Table 1 as MCS/CM/EM/RCS/SM), crosslinking type, bioprinter type, type of extrusion technique (pneumatic, mechanical, piston, screw), printing parameters (resolution, speed), cell source, cell viability and target tissue. Also noted were study authors, study design (*in vitro*/*in vivo*) and year of publication.

## Results

### Types of Bioinks Used for Extrusion Bioprinting

A total of 9,720 studies were identified, 123 of which met inclusion criteria, consisting of a total of 58 reports using natural biomaterials, 26 using synthetic biomaterials and 39 using a combination of biomaterials as bioinks. Alginate (*n* = 53) and PCL (*n* = 30) are the most common types of bioink, followed by gelatin (*n* = 18) and methacrylated gelatin (GelMA) (*n* = 16) (Figure 3).

**Figure 4:**
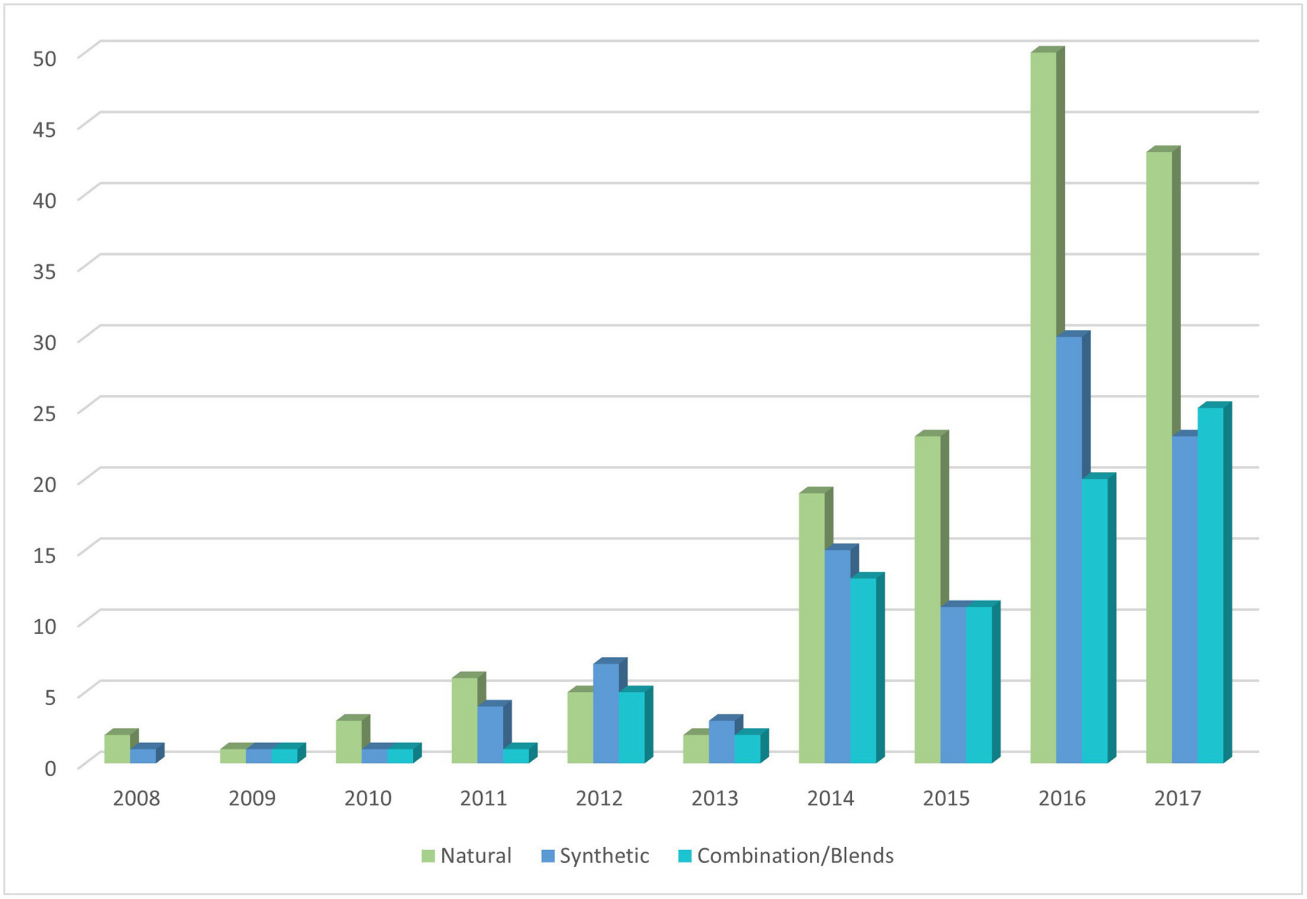
Bioink type frequency in relation to the year of study.

Improvements in technology and biomaterial availability have resulted in an upward trend in the number of studies using bioinks for extrusion. There is a marked increase in the use of all three bioink types (natural, synthetic, and blend) published between 2013 and 2014 and to a lesser degree between 2015 and 2016 (Figure 4). There has been a disproportional increase in the amount of natural bioinks used in the literature compared to synthetic and blend bioinks suggesting increasing popularity in biomimetic approaches (Figure 4).

Pneumatic extrusion bioprinting techniques were the most common (*n* = 78), followed by piston (*n* = 28). Most studies have focused on bone and cartilage as the target tissue type, although bioprinting of solid organs e.g., liver/heart is gaining traction (Supplementary Table 1).

**Figure 5:**
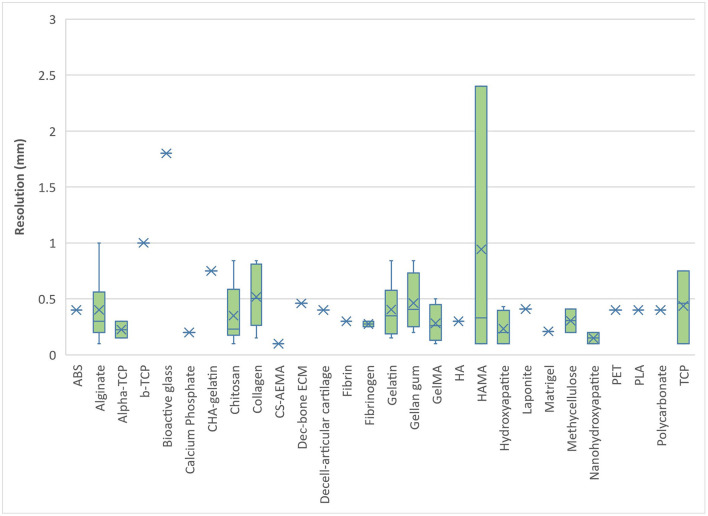
Bioprinting resolution for natural-derived bioinks.

**Figure 6:**
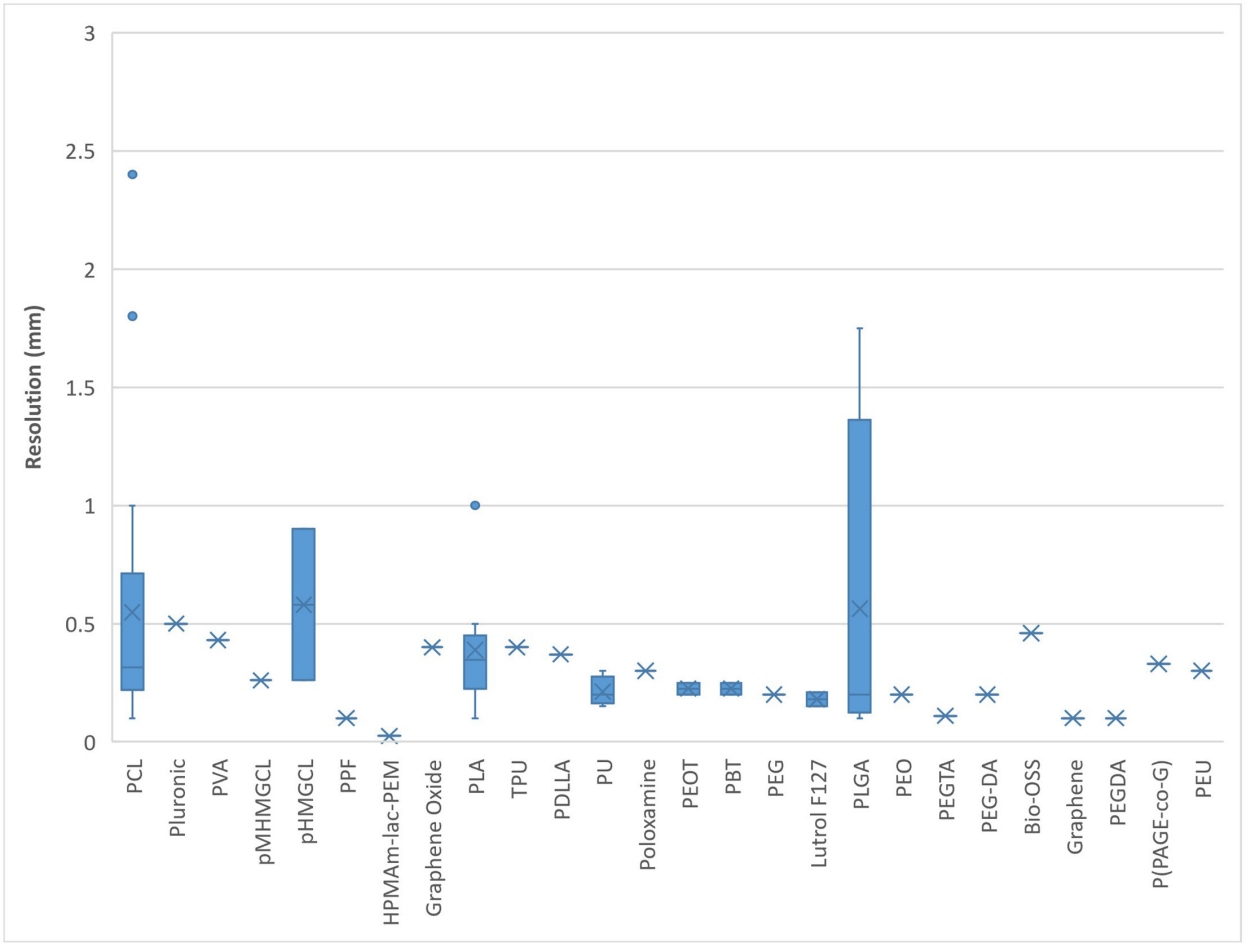
Bioprinting resolution for synthetic-derived bioinks.

Most natural bioinks printed within 1.0 mm resolution. Alginate and HAMA were found to demonstrate the greatest range of printing resolution possible with natural bioinks (0.1–2.4 mm), followed closely by gelatin and collagen (Figure 5). Most synthetic bioinks printed within 0.5 mm resolution except for PCL, pHMGCL and PGLA which demonstrated the greatest range (0.1–1.7 mm) (Figure 6). The lowest resolution printed over all the bioinks (natural, synthetic, or blends) was with a synthetic material; HPMAm-lac-PEM at 0.025 mm.

**Figure 7:**
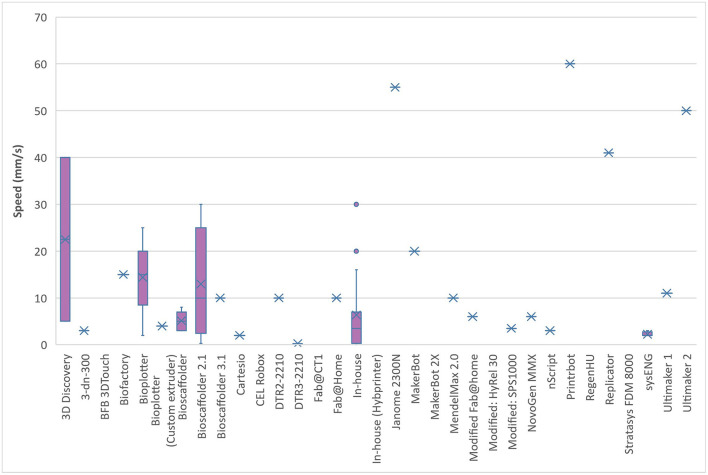
Bioprinting speed for different models of commercial bioprinters.

**Figure 8:**
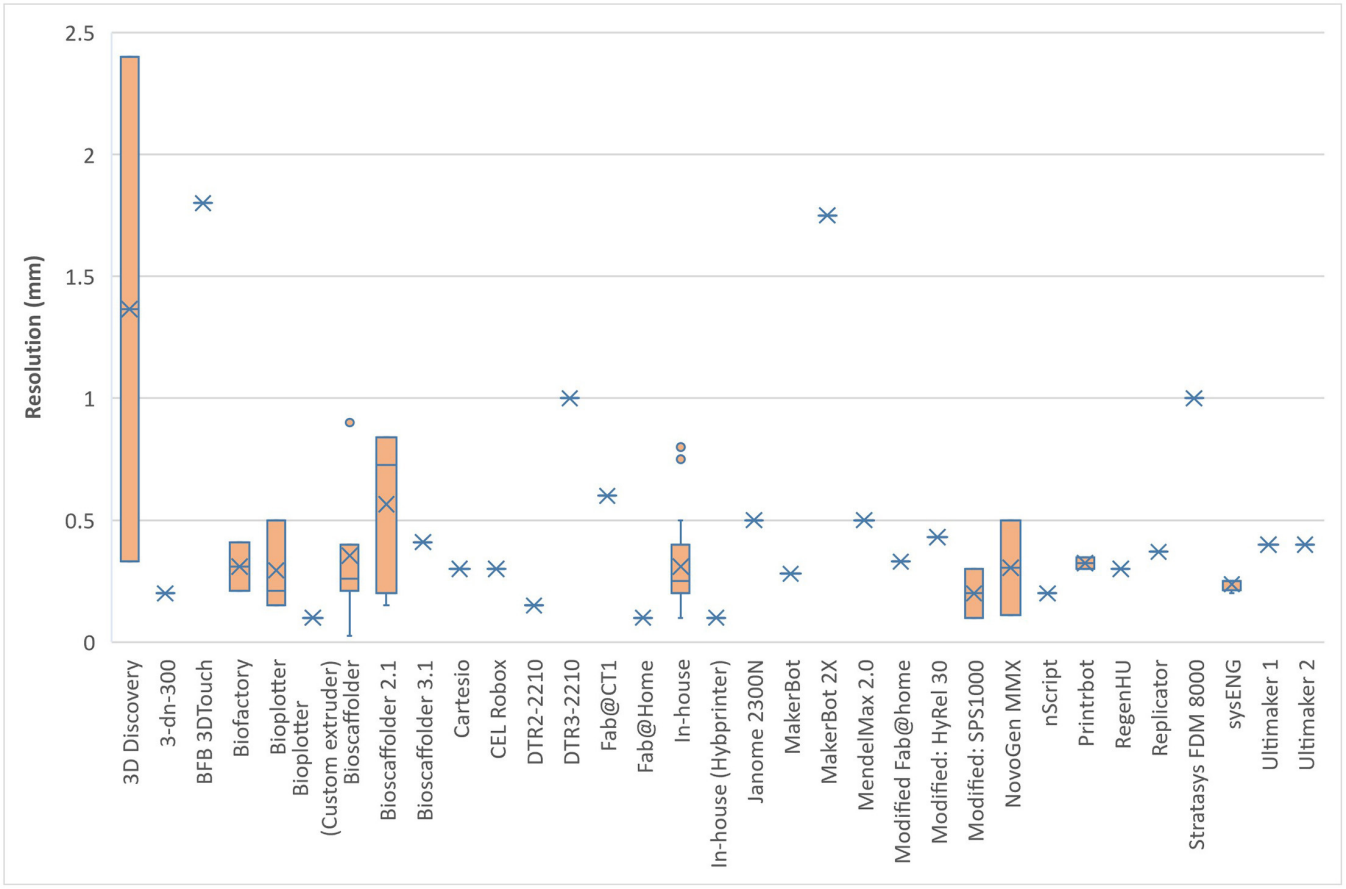
Bioprinting resolution for different models of commercial bioprinters.

From the 123 papers that were analysed, 36 (29%) were in-house printers and 87 (71%) were commercial variations. The analysis of the printing speed and resolution in relation to the bioprinter model used showed a broad scope and range (Figures 7, 8). This is likely to reflect differences in researcher preferences and technical limitations of the different printers used: even the same bioprinter model has varying ranges in both speed and resolution. The 3D Discovery displayed the greatest range of bioprinter speed and resolution of all the models included in this review. However, this is also affected by the tissue type being printed; some cells can only be able to withstand certain printing resolutions and speeds. The lowest printed resolution was the Bioscaffolder at 0.025 mm and the quickest speed at 60 mm/s with the Printrbot (Printrbot, USA).

**Figure 9:**
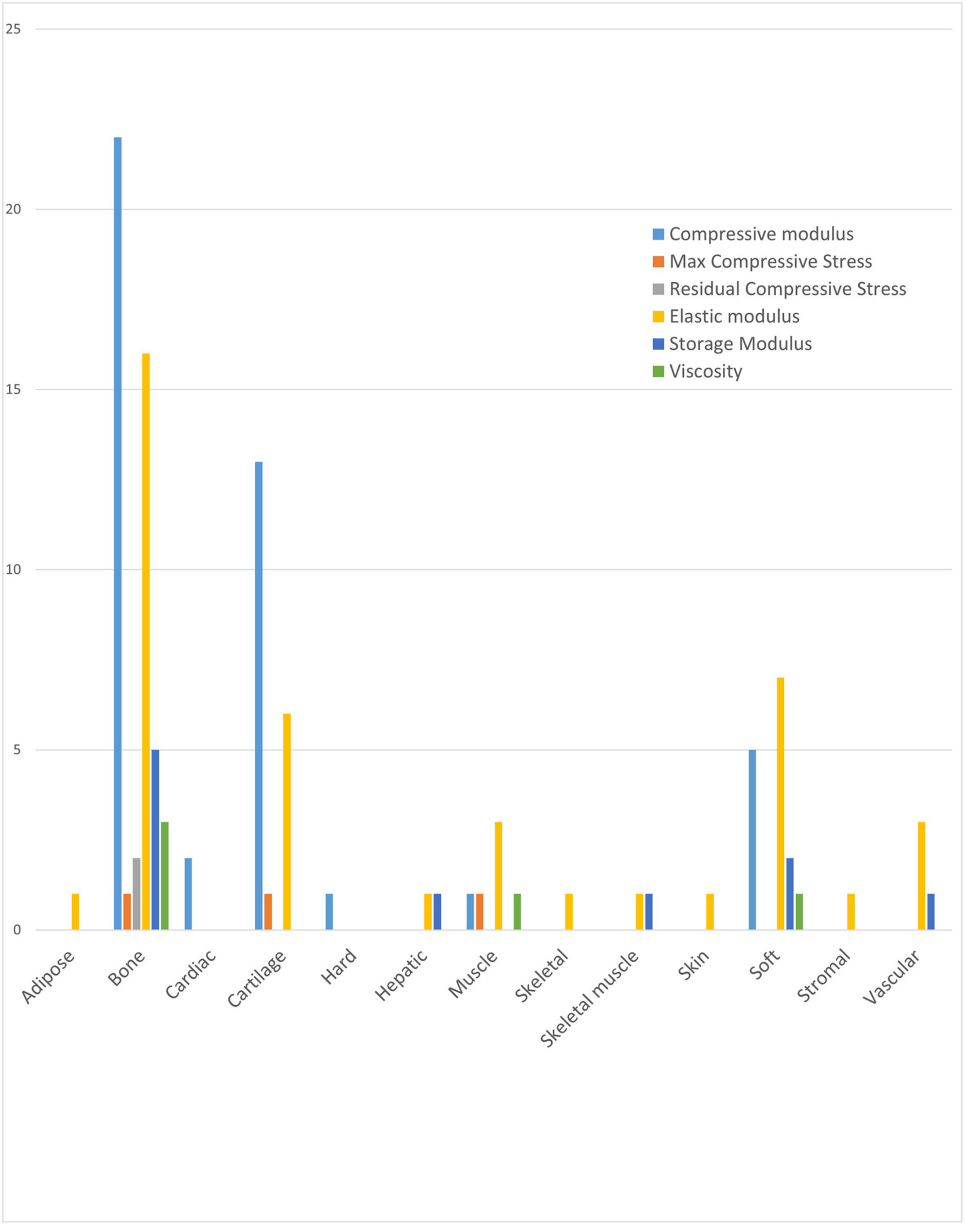
Frequency and class of mechanical assessment for each tissue type.

It was not possible to directly compare biomechanical properties of bioinks used in the literature due to the heterogeneity of mechanical tests used and timing of testing (i.e., pre or post printing, pre or post crosslinking, pre or post tissue maturation). Instead, the most common mechanical moduli used in studies for each tissue type was determined to provide an indication of the most useful biomechanical tests. The results show that compressive modulus was the most frequently applied modulus overall and the most used for bone and cartilage tissues (Figure 9). Elastic modulus was more commonly applied than compressive modulus for a range of soft tissue, including muscle and vascular tissues. Hard tissues within the literature mostly referred to bone and bone derived tissue types (Nikolova and Chavali, 2019). Residual and maximum compressive stress were used least frequently.

## Discussion

Within the last 5 years, development of bioinks have mirrored the exponential growth of research in the 3D bioprinting field. The justification for this growth appears to be 2-fold: firstly the previous two decades have witnessed significant developments in printing technology and stereolithography, secondly, as this systematic review has illustrated, the efforts to engineer and combine novel bioinks have yielded a plethora of printable biomaterials for tissue engineering purposes (Tarassoli et al., 2018b). In line with the expansion of literature in the field of bioink materials and their combination in blends, there has been a call for comprehensive summaries in the field to facilitate the informed design, characterisation and development of further novel bioinks (Ersumo et al., 2016). This systematic review is the first to comprehensively identify the range of biomaterials suitable for extrusion 3D bioprinting, correlate approaches to the different target tissues and evaluate the scope of the methods used to quantify their mechanical properties.

There has been a clear shift towards the use of natural over synthetic scaffolds which may mirror the refinement of natural bioink extraction technology, increased availability and decreased cost. It suggests that research in the field is prioritising biomimicry over customisability. Although synthetic biomaterials were generally found to provide higher printing resolution and allowed tuning of rheological properties through blending (Gopinathan and Noh, 2018), natural bioinks have greater biocompatibility and biodegradability characteristics. Bioinks containing blended biomaterials provide the ability to augment the biological and mechanical properties of one material with another. However, our systematic review has demonstrated that the percentage of published literature using pure bioinks (i.e., not blended or modified beyond the initial extraction/production stage) has more than tripled between 2012 and 2018 which could be attributed to the discovery of novel natural biomaterials with shear thinning properties to allow extrusion as well as improved extraction, chemical modification and crosslinking techniques, enabling individual properties to be maximised. Alginate was the most widely used natural material (*n* = 50), followed by gelatin (*n* = 18) and methacrylated gelatin (GelMA) (*n* = 16), whilst PCL was the most commonly used synthetic material (*n* = 33) for bioink composition. The rationale for alginate use was generally not specified in the included studies, though many did acknowledge that alginate was “the most abundant biomaterial in current bioprinting techniques” (Hill et al., 2006; Lee et al., 2013). Alginate has been widely used in the literature, as a matrix designed for cell encapsulation, due to its versatile, facile, and rapid crosslinking and ability to be mass-produced on a large scale at low cost (Purcell et al., 2009). Fifty litres of 2% alginate solution can be produced from 1 kg alginate, costing < £100 to purchase (SigmaAldrich, 2021a). Compared to alternative natural bioinks such as hyaluronic acid (£57 per gramme) (SigmaAldrich, 2021b). Chemical crosslinking enables gelation within seconds providing an ideal environment for cell storage, proliferation, and differentiation (Galateanu et al., 2012). PCL, on the other hand, has been used predominantly as a mechanically supportive bioink and can be blended with a matrix bioink to provide encapsulation as well as superior mechanical stability (Lee et al., 2013). This may explain why PCL was identified as a widely used bioink for cartilage and bone tissue engineering (Steffens et al., 2016; Stichler et al., 2017). Support bioinks tend to be synthetic polymers, like PCL, due to their thermosensitivity (and thus blendable) which provides controlled biodegradation/bioprintability (Kundu et al., 2015). This thermosensitivity can be taken advantage of by a modification of extrusion printing called “melt blending technology”; polymers that have good mechanical properties with moderate melt viscosity can be blended with other similar polymers in heated extruders (Jiao et al., 2019). PCL has also been found to be effective in both neural (Lee et al., 2017) and hepatic (Lee et al., 2016) tissue engineering, which require vastly different cell culture and bioprinting techniques. This versatility also justifies its popularity as a bioink in the literature. PCL bioprinting can also be combined with other techniques such as electrospinning (Lee et al., 2017) further illustrating its versatility in the field of bioengineering.

Our systematic review revealed that one of the major limitations of most extrusion bioprinting studies is opting for one or two specific bioinks with the goal of engineering a specific tissue type rather than side by side comparisons of bioinks to assess their overall properties and suitability. Akkineni's group (Akkineni A. et al., 2016; Akkineni A. R. et al., 2016) used alginate in 4 separate studies solely for engineering cartilage tissue. For the purposes of their studies, their specific aim was achieved, but it provides limited information on printability and biocompatibility of alginate bioinks overall. Similarly, Dubbin et al. (2017) and Fedorovich et al. (2008, 2009, 2011a,b) assessed synthetic-natural blends (PEGDA and Lutrol F127, respectively) for bone bioprinting using only certain cell types as well thus limiting the potential for full appraisal of those biomaterials. Mesenchymal stem cells appeared to be most commonly used cell source: a product of the dominance of bone and cartilage tissue engineering studies and the relative ease of obtaining mesenchymal stem cells over alternative stem cell sources.

The availability and cost effectiveness of bioprinters has improved significantly in the past few years (Tarassoli et al., 2018a) reflected in the range of bioprinters used in the studies we analysed (Figures 7, 8). There is no “gold standard” bioprinter or extrusion technique and the increasing versatility and customisability of the printers provides a wider capacity for research. Pneumatic force was the most commonly used extrusion technique in those studies that identified the mode of extrusion (64% or 78 out of 123 studies) and suitable for both natural, synthetic and blend bioinks (Liu et al., 2017), followed by piston force technique (*n* = 28). The Bioscaffolder (GeSiM, Germany), 3D Discovery (regenHU, Switzerland) and Bioplotter (EnvisionTEC, Germany) were the most commonly used commercial bioprinters (*n* = 35), but interestingly groups most often opted to create their own in-house devices (*n* = 20). This is most likely due to the low cost of producing such a printer (much of the software and firmware used is open-source; Reid et al., 2016) and the associated potential limitless customisability. The printing speeds ranged from between 2 and 60 mm/s and printing resolutions ranged from 0.025–2.4 mm in the studies that had a full complement of printing information, with no discernible differences between commercial or in-house devices.

**Figure 10:**
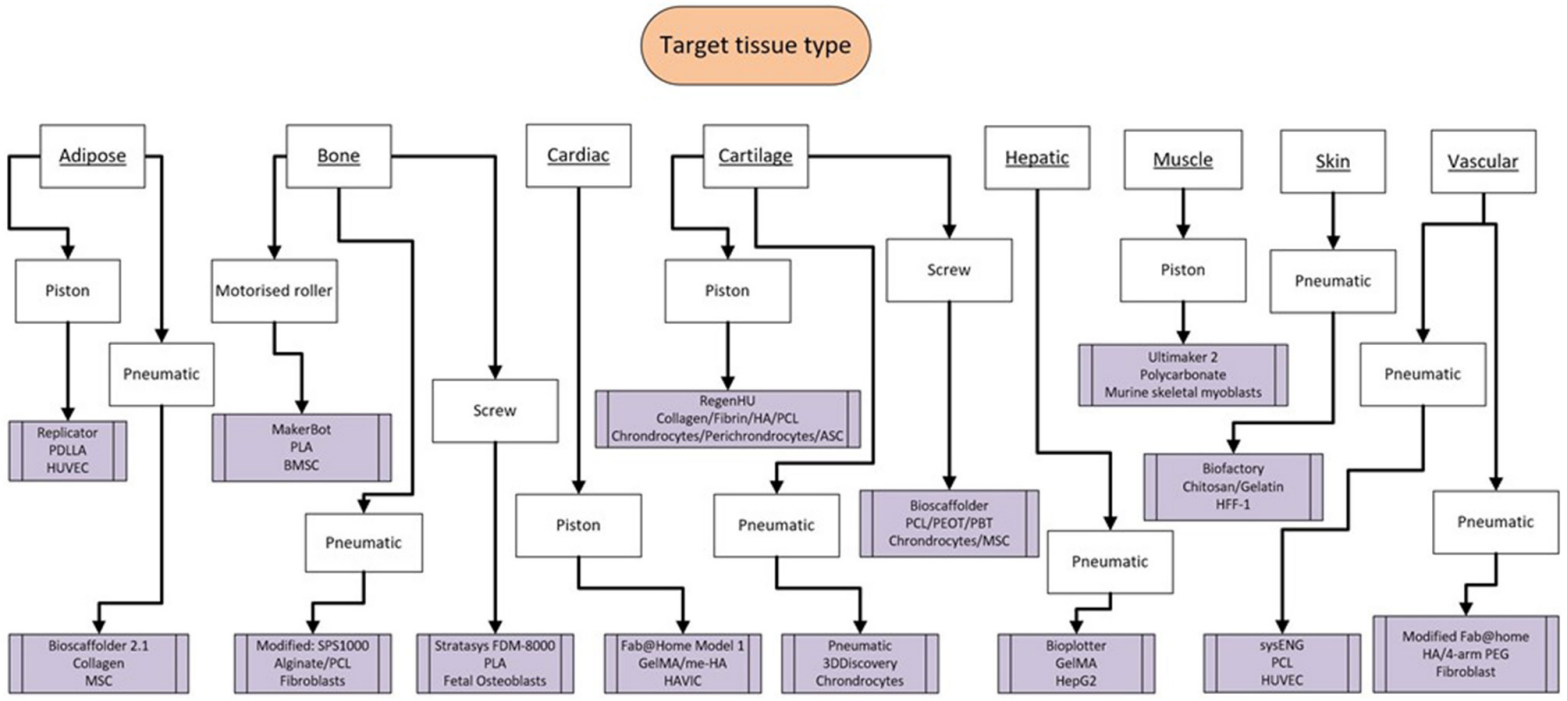
Flowchart highlighting the scope of tissue type produced from the studies alongside the variability of bioprinter model, bioink, and cell source.

We found huge variability in the types of biomechanical tests used to characterise bioinks (Hu et al., 2019) thereby prohibiting direct comparisons of numerical values for moduli. Different approaches for characterising the mechanical properties of bioinks often reflected the target tissue type i.e., compressive tests were more commonly used to assess biomaterials applied to bone and cartilage tissue engineering whereas shear storage modulus was more commonly used to assess biomaterials for engineering soft tissues such as muscle (Figure 10). Many studies also failed to specify whether the mechanical data reflected pre-, during or post-printing, pre- or post-crosslinking and with or without cells (154) (Hegewald et al., 2009; Zadpoor, 2017). It is recognised that biochemical makeup of a material inherently changes after cross-linking, incorporation of cells or secretion of extracellular matrix, which in turn alters its biomechanical properties (Hu et al., 2019) and this needs to be taken into account when characterising bioinks for extrusion. The biomechanical properties required for extrusion such as shear thinning to allow the material to flow with minimal stress to cells, are different to those post-crosslinking or following a period of cellular differentiation and matrix secretion, which should mirror mechanical properties of target tissue type (10, 15).

### Emerging Future Trends From Recent Literature

A further search was carried out to include the literature from 2019 to 2021 using the PRISMA guidelines (Moher et al., 2015) and the same inclusion and exclusion criteria to allow us to establish the emerging recent trends in extrusion 3D bioprinting literature and compare and contrast this to our findings from the previous 12 years (2006–2018). A total of 104 papers met the inclusion criteria over the last 2 years, which is 85% of the total found from the inception of the technology to 2018. This demonstrates an exponential rise of research in the field. The reasons for this increase are multifactorial and likely due to the open-source nature of the technology (i.e., the feasibility of developing an in-house printer with easily available software), automation and customisability of laboratory-based tissue engineering research, combined with the increased availability of high-level work and increasing hubs of expertise in the field.

From analysis of the recent data (2019–2021), alginate continues to be the most widely used bioink accounting for 30% of the literature in pure or blended form. This, however, has reduced from 41% alginate use in literature published between 2006 and 2018 which may be attributed to the emergence of better manipulated synthetic bioinks such as PCL (Diaz-Gomez et al., 2020; Nulty et al., 2021) as well as novel natural bioinks such as nanocellulose (Markstedt et al., 2015; Kyle et al., 2018; Jessop et al., 2019). The most obvious trend that was found was the increase in the tissue type aimed to be recreated. It appears that there has been a shift from the soft tissue to hard and vascular derived tissue. Almost 50% of the studies were recreating bone or vessel tissue types. A hypothesis for this is the development and discovery of various growth factors (Dreyer et al., 2021) as well as more efficient cross-linking techniques to further the accuracy and precision of the printed construct (Benwood et al., 2021; Cooke and Rosenzweig, 2021; Huang et al., 2021).

The techniques of extrusion bioprinting has not changed dramatically in the last 2 years, but there is something to be said for more readily available commercial printers that are capable of high printing resolution and speeds; features that were only previously possible with industry produced equipment (Chang et al., 2021).

## Conclusion

This systematic review provides a snapshot of the current natural and synthetic biomaterials that have been demonstrated to be suitable for extrusion bioprinting different tissue types. There is enormous breadth of bioink, bioprinter, and cell types as well as a range in biomechanical assessments. In part, this breadth can be attributed to the spectrum of tissue types the bioprinting process aims to replicate as well as an indicator that this research field is still in its infancy. There is increasing understanding that development of bioinks needs to address both biological properties, to support cell survival, proliferation, and differentiation, as well as biomechanical properties, to enable dispersion initially as a printed gel, and thereafter as a robust and durable solid tissue. Future research will need more transparency on the biomechanical properties required for different stages of the bioprinting process (pre-printing, shear thinning, post-crosslinking, and post-ECM formation) as well as head-to-head comparisons of different bioinks. The biomechanical suitability of the bioprinted tissue end-products will need to be assessed in the context of native tissue biomechanics and clinical end use application. Appropriate biomechanical methods that best assess *in vivo* stress requirements will need to be established to allow direct comparisons of bioink types. In addition, considering that the inclusion criteria for this systematic review meant that only studies involving cells in combination with biomaterials were included, it was surprising to find that more half of the studies analysed did not assess cell viability explicitly not to mention effects of proliferation or differentiation of bioinks on different cell types. There have been incremental developments in the field over the past 10 years, and the further refinement of bioprinting processes and bioink optimisation holds exciting potential for revolutionising tissue engineering approaches. It is difficult to speculate on what the landscape will be in several years of a decade, but the current frame within the literature is trending towards accuracy as well as precision (Bisht et al., 2021). The field of bioprinting will not be a “on size fits all” approach and unlikely to become a large-scale production of the technology, but rather a bespoke technology that will be produced depending on the patient's needs as well as the clinical application (Alcala-Orozco et al., 2021; Ji and Guvendiren, 2021).

## Data Availability Statement

The original contributions presented in the study are included in the article/Supplementary Material, further inquiries can be directed to the corresponding author/s.

## Author Contributions

ST: majority of writing and inception of review. ZJ: writing and inception of review. TJ: writing. KH: study design and draft work. IW: oversaw project. All authors contributed to the article and approved the submitted version.

## Funding

This work was supported by Medical Research Council (MR/N002431/1).

## Conflict of Interest

The authors declare that the research was conducted in the absence of any commercial or financial relationships that could be construed as a potential conflict of interest.

## Publisher's Note

All claims expressed in this article are solely those of the authors and do not necessarily represent those of their affiliated organizations, or those of the publisher, the editors and the reviewers. Any product that may be evaluated in this article, or claim that may be made by its manufacturer, is not guaranteed or endorsed by the publisher.

## Abbreviations

ABS: Acrylonitrile butadiene styrene
ADSC: Adipose Derived Stem Cell
ASSMC: Aortic sinus smooth muscle cells
BMPC: Bone marrow plasma cell
B-TCP: Beta tricalcium phosphate
CM: Compressive Modulus
ECM: Extracellular matrix
EM (Compressive): Elastic Modulus
EPC: Endothelial progenitor cell
HAMA: Hyaluronic Acid Methyacrylate
HAVIC: Human aortic valve interstitial cell
HDMEC: Human Brain Microvascular Endothelial cell
htMSC: Human turbinate mesenchymal stem cells
HUCPVC: Human Umbilical Cord Perivascular Cells
HUVEC: Human umbilical vein endothelial cell
MCS: Maximum Compressive Strength
MSC: Mesenchymal stem cell
P(AGE-co-G): Ally-functionalised poly(glycidol)s
PAEC: Porcine aorta endothelial cells
PBT: Polybutylene terephthalate
PCL: Polycaprolactone
PDLLA: Poly-DL-lactic acid
PEG-DA: Polyethylene glycol diacrylate
PEOT: Poly(ethylene oxide terephthalate)
PEU: Poly ether urethane
PGS: Pyrolytic graphite sheet
pHMGCL: Phenyl magnesium chloride
PLGA: Poly-lactic-co-glycolic acid
PPF: Polypropylene fumarate
PU: Polyurethane
PUPEO: Polyurethane Polyethylene oxide
PVA: Polyvinyl acetate
RCS: Residual Compressive Stress
SM: Shear Storage Modulus
SEM: Shear Elastic Modulus
SVF: Stromal vascular fraction
TPU: Thermoplastic polyurethane
V: Viscosity.
