# Supplementary material for: Candidate Bioinks for Extrusion 3D Bioprinting—A Systematic Review of the Literature
Source: Front Bioeng Biotechnol. 2021 Oct 13;9:616753. doi: 10.3389/fbioe.2021.616753 (PMC8548422; doi:10.3389/fbioe.2021.616753)
Supplement: Supplementary file 1 [file Table_1.docx]

**Table 1.** Summary bioink types used for bioprinting different tissue types.

| **Tissue** | **Bioink** | **Bioink type** | **Extrusion subtype** | **Cell type** | **Cell species** | **Cell viability** | **Type of biochemical assessment** | **Range of properties (MCS/CM/EM/RCS/SM – kPA – Pa s)** | **Reference** |
| --- | --- | --- | --- | --- | --- | --- | --- | --- | --- |
| **Adipose** | PDLLA | Synthetic | Piston | HUCPVC, HUVEC | Human | N/a | EM | 1200 | [1] |
|  | Collagen | Natural combination | Pneumatic | MSC | Human | N/a | CM | 48 | [2] |
| **Bone** | Alginate | Natural combination | Pneumatic | MSC; MSC and chondrocytes | Goat; Human | 80; 85 | EM | 7.6 | [3, 4] |
|  | Alginate  Gelatin | Natural combination | Pneumatic | EPC; MSC | Human | N/a | CM; EM | 2.5x increase after crosslinking; 25000 | [5, 6] |
|  | Alginate β-TCP | Natural combination | Piston | Osteoblasts | Human | 80 | V | 0.15 | [7] |
|  | Alginate Gelatin | Natural combination | Pneumatic | Osteoblast-like cells | Human | N/a | - | - | [8, 9] |
|  | Alginate Gellan Calcium Phosphate; Alginate Laponite Methycellulose | Natural combination | Pneumatic | HDMEC; MSC | Human | N/a; 70 | CM; EM | 40; 18 | [10, 11] |
|  | Alginate hydroxyapatite | Natural combination | Pneumatic | CCL-226 | Murine | N/a | - | - | [12] |
|  | α-TCP Alginate | Natural combination | Pneumatic | Pre-osteoblasts | Murine | N/a | - | - | [13] |
|  | Chitosan; Chitosan Gelatin; Chitosan Hydroxyapatite | Natural combination | Pneumatic | Osteoblasts; ADSC; Pre-osteoblasts | Human | N/a; N/a: 91 | EM; SM | 10-15; 874 | [14-16] |
|  | Gelatin Magnesium Phosphate | Natural combination | Piston | Fibroblasts | Human | N/a | CM | 250 -50000 | [17] |
|  | GelMA | Natural | Piston | HUVEC and MSC; Osteoblast-like cells | Human | 93; N/a | CM; SM | 6.5; 1 | [18, 19] |
|  | Matrigel Lutrol F127 | Synthetic combination | Pneumatic | MSC | Goat | 45 | EM | 21 | [20] |
|  | TCP PLGA; TCP PCL PLGA | Synthetic combination | Piston; Pneumatic | MSC | Rabbit | N/a | CM; EM | 4800-53100; 18000 | [21, 22] |
|  | nanohydroxyapatite PEG-DA | Natural/Synthetic blend | N/a | MSC | Human | N/a | EM | 8000 | [23] |
|  | Alginate Pluronic | Natural/Synthetic blend | Piston | MSC | Human | 87 | - | - | [24] |
|  | Chitosan Eggshell derived Calcium phosphate | Natural combination | Pneumatic | MSC | Human | N/a | MCS | 9500 | [25] |
|  | Alginate PLGA | Natural/Synthetic blend | Pneumatic | MSC and HEK293 | Human | N/a | CM | 1.5 | [26] |
|  | Alginate hydroxyapatite PVA; Alginate PVA | Natural/Synthetic blend | Pneumatic | MC3T3-E1; MSC | Murine | 98; N/a | CM | 9 | [27, 28] |
|  | nanohydroxyapatite PCL PPF | Natural/Synthetic blend | N/a | MC3T3-E1 | Murine | N/a | CM; EM | 38000; 3000 | [29] |
|  | Alginate PCL | Natural/Synthetic blend | Pneumatic | MSC; Fibroblasts | Porcine | 70; 89 | CM; EM | 1600; 42000 | [30-32] |
|  | TCP PCL; Decellularised-bone ECM PCL; PCL Bio-OSS | Synthetic combination | Pneumatic | ASC | Human | N/a | CM | 350000 | [33] |
|  | HAMA P(PAGE-co-G) | Natural/Synthetic blend | Pneumatic | MSC | Human/equine | N/a | EM | 50 | [34] |
|  | Hydroxyapatite PEU | Natural/Synthetic blend | N/a | Preosteoblast cells | Human | N/a | CM | 50000 | [35] |
|  | Alendronate releasing PCL | Synthetic | Pneumatic | Osteoblast-like cells | Human | N/a | - | - | [36] |
|  | Lutrol F127 | Synthetic | Pneumatic | MSC | Goat | 95 | - | - | [20] |
|  | PCL | Synthetic | N/a; N/a; Screw | MSC | Human | N/a | EM; SM | 50000; 18000 | [37-39] |
|  | PCL PLGA | Synthetic combination | Pneumatic | hTMSC | Human | N/a | - | - | [40] |
|  | PCL Poloxamine | Synthetic combination | Piston | ADSC | Human | N/a | CM | 12000 | [41] |
|  | pHMGCL | Synthetic | Screw | MSC | Human | N/a | - | - | [37] |
|  | PLA | Synthetic | Piston; Screw | Osteoblasts | Murine | N/a | CM; EM | 500000; 3000000 | [42, 43] |
|  | β-TCP PCL | Natural/Synthetic blend | N/a | BMPC | Human | N/a | - | - | [44] |
| **Bone/Cartilage** | CHA-gelatin TCP PCL | Natural/Synthetic blend | Screw | MSC | Human | N/a | CM | 82000 | [45] |
|  | PCL | Synthetic | Motorised roller; Screw | MSC | Human | N/a | CM | 32000 | [46] |
|  | PLA | Synthetic | Motorised roller; Screw | MSC | Human | N/a | CM; EM | 26000; 31000 | [46-48] |
|  | α-TCP collagen; Alginate PCL | Natural/Synthetic blend | Pneumatic | Fibroblasts; Osteoblast-like cells | Human | 90; 95 | EM; MCS | 550-5800; 460 | [49, 50] |
|  | Calcium Phosphate PLA PEG | Natural/Synthetic blend | Pneumatic | MSC | Rat | N/a | CM | 100000 | [51] |
|  | PEOT PBT | Synthetic combination | Screw | MSC | Human | N/a | - | - | [47] |
| **Cardiac** | Alginate | Natural | N/a | PAEC | Porcine | N/a | - | - | [52] |
|  | Alginate GelMA | Natural combination | N/a | HUVEC | Human | 75 | EM | 55 | [53] |
|  | GelMA me-HA | Natural combination | Piston | HAVIC | Human | 90 | CM | 12 | [54] |
|  | Alginate GelMA PEGDA | Natural/Synthetic blend | Piston | ADSC, HAVIC and ASSMC | Human | 80 | CM | 120 | [55] |
|  | PU | Synthetic | Pneumatic | Cardiac progenitor cells | Human | 95 | EM | 10000 | [56] |
| **Cartilage** | Alginate | Natural | Pneumatic | Endothelial cells; chondrocytes | Rat | N/a; 80 | CM | 70 | [57, 58] |
|  | Alginate with Chitosan, or collagen, or gelatin, or gellan | Natural combination | Pneumatic | HDMEC | Human | 85 | EM | 750 – 3000 | [59] |
|  | Alginate Gellan | Natural combination | Pneumatic | Chondrocytes; MSC | Bovine | 94; 85 | CM; EM; V | 3000; 230; 800 | [60, 61] |
|  | Alginate GelMA CS-AEMA HAMA | Natural combination | Pneumatic | MSC | Human | 90 | CM | 100 | [62] |
|  | Decellularised- articular cartilage Collagen | Natural combination | Pneumatic | Chondrocytes | Rabbit | N/a | CM; V | 89; 1800 | [63] |
|  | GelMA Gellan gum | Natural combination | Pneumatic | MSC; chondrocytes | Equine | 94; N/a | CM; EM; V | 5; 77; 0.075 | [64, 65] |
|  | GelMA HAMA | Natural combination | Piston | MSC | Human | 97 | EM | 480 | [66] |
|  | GelMA PCL pMHMGCL | Natural/Synthetic blend | Pneumatic | Chondrocytes | Human | N/a | RCS | 7.5 – 14.1 | [67] |
|  | Collagen Fibrin HA PCL | Natural/Synthetic blend | Piston | Chondrocytes Perichondrocytes ASC | Goat | N/a | EM | 6 | [68] |
|  | Alginate Nanocellulose | Natural combination | Pneumatic | Chondrocytes and MSC | Bovine | N/a | MCS | 46 | [69] |
|  | Alginate PLA | Natural/Synthetic blend | Pneumatic | Fibroblasts; Chondrocytes | Murine | 91; 80 | EM | 25 | [70, 71] |
|  | HAMA PCL | Natural/Synthetic blend | Pneumatic | Chondrocytes | Equine | N/a | EM | 4000 | [65] |
|  | HPMAm-lac-PEG | Synthetic | Pneumatic | Chondrocytes | Equine | 90 | EM | 119 | [72] |
|  | PCL | Synthetic | Piston | MSC | Rabbit | N/a |  |  | [73] |
|  | PCL PLA; PCL PLGA | Synthetic combination | Pneumatic | Fibroblasts | Rabbit | 97 | CM | 18000 – 38000 | [74] |
|  | PEOT PBT | Synthetic combination | Screw | MSC | Rat | N/a | - | - | [75] |
|  | PLGA; PU; PUPEO | Synthetic combination | Pneumatic | Chondrocytes | Rat | N/a | CM | 400 – 1660 | [76] |
|  | dex-HEMA | Natural | Pneumatic | Chondrocytes | Equine | N/a | SM | 15 | [77] |
| **Hepatic** | GelMA | Natural | Pneumatic | HepG2 | Human | 97 | - | - | [78] |
|  | Gelatin HA PEGDA | Natural/Synthetic blend | N/a | Hepatocytes Stellate cells | Human | N/a | EM | 2000 | [79] |
| **Muscle** | Alginate Gelatin | Natural combination | Pneumatic | Myoblasts | Murine | 96 | CM | 7 | [80] |
|  | Fibrinogen Gelatin HA PU | Natural/Synthetic blend | N/a | Fibroblasts and Myoblasts | Murine | N/a | SEM | 390 | [81] |
|  | ABS; PET; PLA; Polycarbonate | Natural/Synthetic blend | Piston | Myoblasts | Murine | N/a | - | - | [82] |
|  | Alginate Fibrinogen | Natural combination | Pneumatic | Muscle precursor cells | Murine | N/a | SEM | 48 | [83] |
|  | Decellularised-skeletal muscle ECM PCL | Natural/Synthetic blend | Pneumatic | Myoblasts | Murine | 90 | SEM | 12 | [84] |
| **Neural** | Collagen | Natural | Pneumatic | Neural stem cells | Murine | 93 | - | - | [85] |
|  | Alginate | Natural | Piston | Glioma U87 | Human | 88 | EM | 28 | [86] |
|  | Graphene PCL | Natural/Synthetic blend | Pneumatic | PC12 | Rat | N/a | EM | 5600 | [87] |
| **Other Soft Tissue** | Chitosan Gelatin | Natural combination | Pneumatic | HFF-1 | Human | N/a | - | - | [88] |
|  | Alginate Collagen | Natural combination | Pneumatic | MG63 ASC | Human | 95 | EM; SM | 300; 3 | [89] |
|  | Alginate Methylcellulose | Natural combination | Pneumatic | MSC | Human | 75 | CM | 72 | [90] |
|  | Chitosan | Natural | Pneumatic | Fibroblasts | Human | N/a | - | - | [91] |
|  | HA | Natural | Screw; N/a | Fibroblasts | Murine | 90; 80 | EM | 20 | [92, 93] |
|  | P1 and P2 peptide tethered alginate | Natural | Pneumatic | Fibroblasts | Murine | 98 | SM | 4 | [94] |
|  | Alginate PCL | Natural/Synthetic blend | Pneumatic | C20A4 | Human | 70 | EM | 6000 | [95] |
|  | Bioactive glass PCL | Synthetic | Screw | Fibroblast | Human | N/a | CM | 150000 | [96] |
|  | Soy protein isolate PCL-based PU PLLA | Natural/Synthetic blend | Pneumatic | Fibroblasts (L929) | Murine | 100 | - | - | [97] |
|  | Alginate PEGDA | Natural/Synthetic blend | Pneumatic | Fibroblasts | Murine | 96 | SM | 8 | [94] |
|  | Xanthan gum PEGDA | Natural/Synthetic blend | Pneumatic | Fibroblasts | Murine | 96 | SM | 12 | [94] |
|  | Graphene Oxide PLA TPU | Natural/Synthetic blend | Piston | Fibroblasts | Murine | N/a | CM | 140000 | [98] |
|  | PEG | Synthetic | N/a | MSC | Murine | N/a | V | 4.5 | [99] |
|  | PGS | Synthetic | Piston | Fibroblasts | Murine | N/a | EM | 430 | [100] |
|  | PLA | Synthetic | Motorised roller | Preosteoblast cells | Murine | N/a | - | - | [101] |
|  | PLGA | Synthetic | Pneumatic | Lung fibroblasts; ADMSC | Human | N/a | CM | 150000 | [102, 103] |
|  | Pluronic | Synthetic | Piston | SVF cells | Rat | 68 | - | - | [104] |
|  | PU (ester urethane) | Synthetic | Motorised roller | Fibroblasts | Rat | N/a | - | - | [105] |
|  | Gelatin | Natural | Piston | MSC Fibroblasts | Human | N/a | - | - | [106] |
| **Skin** | Alginate Gelatin | Natural combination | N/a; N/a; N/a; Piston | ESC; epithelial cells; fibroblasts | Murine | 80; N/a; 55; N/a | SM | 230 | [107-110] |
|  | Alginate Collagen Gelatin | Natural combination | N/a | Corneal epithelial cells | Human | 94 | - | - | [111] |
| **Vascular** | Alginate GelMA | Natural combination | Pneumatic | HDF HUVEC HepG2 MSC | Human | 80 | - | - | [112] |
|  | HA 4-arm PEG | Natural/Synthetic blend | N/a | Fibroblast | Human | N/a | - | - | [113] |
|  | GelMA | Natural | Pneumatic | HUVEC; fibroblasts | Human | N/a; 95 | SM | 11 - 590 | [94, 114] |
|  | GelMA PEGTA | Natural/Synthetic blend | Pneumatic | HUVEC and MSC | Human | 80 | CM | 50 | [115] |
|  | Alginate PEGDA | Natural/Synthetic blend | Pneumatic | HUVEC Fibroblasts HepG2 | Human | 95 | EM | 600 | [116] |
|  | PCL | Synthetic | Pneumatic | HUVEC | Human | N/a | - | - | [117] |
|  | PCL PEGDA | Synthetic combination | Piston | HUVEC and MSC | Human | 90 | EM | 6000 | [118] |

## **References**

[1] Chhaya M P, Melchels F P, Holzapfel B M, Baldwin J G and Hutmacher D W 2015 Sustained regeneration of high-volume adipose tissue for breast reconstruction using computer aided design and biomanufacturing *Biomaterials* **52** 551-60

[2] Lode A, Meyer M, Bruggemeier S, Paul B, Baltzer H, Schropfer M, Winkelmann C, Sonntag F and Gelinsky M 2016 Additive manufacturing of collagen scaffolds by three-dimensional plotting of highly viscous dispersions *Biofabrication* **8** 015015

[3] Fedorovich N E, De Wijn J R, Verbout A J, Alblas J and Dhert W J 2008 Three-dimensional fiber deposition of cell-laden, viable, patterned constructs for bone tissue printing *Tissue engineering. Part A* **14** 127-33

[4] Fedorovich N E, Kuipers E, Gawlitta D, Dhert W J and Alblas J 2011 Scaffold porosity and oxygenation of printed hydrogel constructs affect functionality of embedded osteogenic progenitors *Tissue engineering. Part A* **17** 2473-86

[5] Poldervaart M T, Gremmels H, van Deventer K, Fledderus J O, Öner F C, Verhaar M C, Dhert W J A and Alblas J 2014 Prolonged presence of VEGF promotes vascularization in 3D bioprinted scaffolds with defined architecture *Journal of Controlled Release* **184** 58-66

[6] Pan T, Song W, Cao X and Wang Y 2016 3D Bioplotting of Gelatin/Alginate Scaffolds for Tissue Engineering: Influence of Crosslinking Degree and Pore Architecture on Physicochemical Properties *Journal of Materials Science & Technology* **32** 889-900

[7] Diogo G S, Gaspar V M, Serra I R, Fradique R and Correia I J 2014 Manufacture of beta-TCP/alginate scaffolds through a Fab@home model for application in bone tissue engineering *Biofabrication* **6** 025001

[8] Zehnder T, Sarker B, Boccaccini A R and Detsch R 2015 Evaluation of an alginate-gelatine crosslinked hydrogel for bioplotting *Biofabrication* **7** 025001

[9] Leite A J, Sarker B, Zehnder T, Silva R, Mano J F and Boccaccini A R 2016 Bioplotting of a bioactive alginate dialdehyde-gelatin composite hydrogel containing bioactive glass nanoparticles *Biofabrication* **8** 035005

[10] Ahlfeld T, Akkineni A R, Forster Y, Kohler T, Knaack S, Gelinsky M and Lode A 2017 Design and Fabrication of Complex Scaffolds for Bone Defect Healing: Combined 3D Plotting of a Calcium Phosphate Cement and a Growth Factor-Loaded Hydrogel *Ann Biomed Eng* **45** 224-36

[11] Ahlfeld T, Cidonio G, Kilian D, Duin S, Akkineni A R, Dawson J I, Yang S, Lode A, Oreffo R O C and Gelinsky M 2017 Development of a clay based bioink for 3D cell printing for skeletal application *Biofabrication* **9** 034103

[12] Wang Q, Xia Q, Wu Y, Zhang X, Wen F, Chen X, Zhang S, Heng B C, He Y and Ouyang H W 2015 3D-Printed Atsttrin-Incorporated Alginate/Hydroxyapatite Scaffold Promotes Bone Defect Regeneration with TNF/TNFR Signaling Involvement *Advanced healthcare materials* **4** 1701-8

[13] Raja N and Yun H-s 2016 A simultaneous 3D printing process for the fabrication of bioceramic and cell-laden hydrogel core/shell scaffolds with potential application in bone tissue regeneration *Journal of Materials Chemistry B* **4** 4707-16

[14] Liu I H, Chang S H and Lin H Y 2015 Chitosan-based hydrogel tissue scaffolds made by 3D plotting promotes osteoblast proliferation and mineralization *Biomedical materials (Bristol, England)* **10** 035004

[15] Chen H, Liu Y, Jiang Z, Chen W, Yu Y and Hu Q 2014 Cell-scaffold interaction within engineered tissue *Exp Cell Res* **323** 346-51

[16] Demirtas T T, Irmak G and Gumusderelioglu M 2017 A bioprintable form of chitosan hydrogel for bone tissue engineering *Biofabrication* **9** 035003

[17] Farag M M and Yun H-s 2014 Effect of gelatin addition on fabrication of magnesium phosphate-based scaffolds prepared by additive manufacturing system *Materials Letters* **132** 111-5

[18] Byambaa B, Annabi N, Yue K, Trujillo-de Santiago G, Alvarez M M, Jia W, Kazemzadeh-Narbat M, Shin S R, Tamayol A and Khademhosseini A 2017 Bioprinted Osteogenic and Vasculogenic Patterns for Engineering 3D Bone Tissue **6**

[19] McBeth C, Lauer J, Ottersbach M, Campbell J, Sharon A and Sauer-Budge A F 2017 3D bioprinting of GelMA scaffolds triggers mineral deposition by primary human osteoblasts *Biofabrication* **9** 015009

[20] Fedorovich N E, Swennen I, Girones J, Moroni L, van Blitterswijk C A, Schacht E, Alblas J and Dhert W J 2009 Evaluation of photocrosslinked Lutrol hydrogel for tissue printing applications *Biomacromolecules* **10** 1689-96

[21] Chen S H, Wang X L, Xie X H, Zheng L Z, Yao D, Wang D P, Leng Y, Zhang G and Qin L 2012 Comparative study of osteogenic potential of a composite scaffold incorporating either endogenous bone morphogenetic protein-2 or exogenous phytomolecule icaritin: an in vitro efficacy study *Acta biomaterialia* **8** 3128-37

[22] Pati F, Ha D H, Jang J, Han H H, Rhie J W and Cho D W 2015 Biomimetic 3D tissue printing for soft tissue regeneration *Biomaterials* **62** 164-75

[23] Nowicki M A, Castro N J, Plesniak M W and Zhang L G 2016 3D printing of novel osteochondral scaffolds with graded microstructure *Nanotechnology* **27** 414001

[24] Armstrong J P, Burke M, Carter B M, Davis S A and Perriman A W 2016 3D Bioprinting Using a Templated Porous Bioink *Advanced healthcare materials* **5** 1724-30

[25] Dadhich P, Das B, Pal P, Srivas P K, Dutta J, Ray S and Dhara S 2016 A Simple Approach for an Eggshell-Based 3D-Printed Osteoinductive Multiphasic Calcium Phosphate Scaffold *ACS Applied Materials & Interfaces* **8** 11910-24

[26] Do A-V, Akkouch A, Green B, Ozbolat I, Debabneh A, Geary S and Salem A K 2017 Controlled and Sequential Delivery of Fluorophores from 3D Printed Alginate-PLGA Tubes *Annals of Biomedical Engineering* **45** 297-305

[27] Bendtsen S T and Wei M 2017 In vitro evaluation of 3D bioprinted tri-polymer network scaffolds for bone tissue regeneration *Journal of biomedical materials research. Part A* **105** 3262-72

[28] Luo Y, Lode A and Gelinsky M 2013 Direct plotting of three-dimensional hollow fiber scaffolds based on concentrated alginate pastes for tissue engineering *Advanced healthcare materials* **2** 777-83

[29] Buyuksungur S, Endogan Tanir T, Buyuksungur A, Bektas E I, Torun Kose G, Yucel D, Beyzadeoglu T, Cetinkaya E, Yenigun C, Tonuk E, Hasirci V and Hasirci N 2017 3D printed poly(epsilon-caprolactone) scaffolds modified with hydroxyapatite and poly(propylene fumarate) and their effects on the healing of rabbit femur defects *Biomaterials science* **5** 2144-58

[30] Cunniffe G M, Gonzalez-Fernandez T, Daly A, Sathy B N, Jeon O, Alsberg E and Kelly D J 2017 Three-Dimensional Bioprinting of Polycaprolactone Reinforced Gene Activated Bioinks for Bone Tissue Engineering *Tissue engineering. Part A* **23** 891-900

[31] Daly A C, Cunniffe G M, Sathy B N, Jeon O, Alsberg E and Kelly D J 2016 3D Bioprinting of Developmentally Inspired Templates for Whole Bone Organ Engineering *Advanced healthcare materials* **5** 2353-62

[32] Cornock R, Beirne S, Thompson B and Wallace G G 2014 Coaxial additive manufacture of biomaterial composite scaffolds for tissue engineering *Biofabrication* **6** 025002

[33] Nyberg E, Rindone A, Dorafshar A and Grayson W L 2017 Comparison of 3D-Printed Poly-varepsilon-Caprolactone Scaffolds Functionalized with Tricalcium Phosphate, Hydroxyapatite, Bio-Oss, or Decellularized Bone Matrix *Tissue engineering. Part A* **23** 503-14

[34] Stichler S, Bock T, Paxton N, Bertlein S, Levato R, Schill V, Smolan W, Malda J, Tessmar J, Blunk T and Groll J 2017 Double printing of hyaluronic acid/poly(glycidol) hybrid hydrogels with poly(epsilon-caprolactone) for MSC chondrogenesis *Biofabrication* **9** 044108

[35] Yu J, Xu Y, Li S, Seifert G V and Becker M L 2017 Three-Dimensional Printing of Nano Hydroxyapatite/Poly(ester urea) Composite Scaffolds with Enhanced Bioactivity **18** 4171-83

[36] Kim S E, Yun Y P, Shim K S, Kim H J, Park K and Song H R 2016 3D printed alendronate-releasing poly(caprolactone) porous scaffolds enhance osteogenic differentiation and bone formation in rat tibial defects *Biomedical materials (Bristol, England)* **11** 055005

[37] Seyednejad H, Gawlitta D, Dhert W J A, van Nostrum C F, Vermonden T and Hennink W E 2011 Preparation and characterization of a three-dimensional printed scaffold based on a functionalized polyester for bone tissue engineering applications *Acta biomaterialia* **7** 1999-2006

[38] Steffens D, Rezende R A, Santi B, Pereira F D, Inforcatti Neto P, da Silva J V and Pranke P 2016 3D-printed PCL scaffolds for the cultivation of mesenchymal stem cells *Journal of applied biomaterials & functional materials* **14** e19-25

[39] Ostrowska B, Di Luca A, Szlazak K, Moroni L and Swieszkowski W 2016 Influence of internal pore architecture on biological and mechanical properties of three-dimensional fiber deposited scaffolds for bone regeneration *Journal of biomedical materials research. Part A* **104** 991-1001

[40] Shim J H, Kim S E, Park J Y, Kundu J, Kim S W, Kang S S and Cho D W 2014 Three-dimensional printing of rhBMP-2-loaded scaffolds with long-term delivery for enhanced bone regeneration in a rabbit diaphyseal defect *Tissue engineering. Part A* **20** 1980-92

[41] Costa P F, Puga A M, Diaz-Gomez L, Concheiro A, Busch D H and Alvarez-Lorenzo C 2015 Additive manufacturing of scaffolds with dexamethasone controlled release for enhanced bone regeneration *International journal of pharmaceutics* **496** 541-50

[42] Souness A, Zamboni F, Walker G M and Collins M N 2018 Influence of scaffold design on 3D printed cell constructs *Journal of biomedical materials research. Part B, Applied biomaterials* **106** 533-45

[43] Wurm M C, Most T, Bergauer B, Rietzel D, Neukam F W, Cifuentes S C and Wilmowsky C V 2017 In-vitro evaluation of Polylactic acid (PLA) manufactured by fused deposition modeling *Journal of biological engineering* **11** 29

[44] Sharaf B, Faris C B, Abukawa H, Susarla S M, Vacanti J P, Kaban L B and Troulis M J 2012 Three-dimensionally printed polycaprolactone and beta-tricalcium phosphate scaffolds for bone tissue engineering: an in vitro study *Journal of oral and maxillofacial surgery : official journal of the American Association of Oral and Maxillofacial Surgeons* **70** 647-56

[45] Tarik Arafat M, Lam C X F, Ekaputra A K, Wong S Y, He C, Hutmacher D W, Li X and Gibson I 2011 High performance additive manufactured scaffolds for bone tissue engineering application *Soft Matter* **7** 8013-22

[46] Moura C S, Ferreira F C and Bártolo P J 2016 Comparison of Three-dimensional Extruded Poly (ɛ-Caprolactone) and Polylactic acid Scaffolds with Pore size Variation *Procedia CIRP* **49** 209-12

[47] Hendrikson W J, Zeng X, Rouwkema J, van Blitterswijk C A, van der Heide E and Moroni L 2016 Biological and Tribological Assessment of Poly(Ethylene Oxide Terephthalate)/Poly(Butylene Terephthalate), Polycaprolactone, and Poly (L\DL) Lactic Acid Plotted Scaffolds for Skeletal Tissue Regeneration *Advanced healthcare materials* **5** 232-43

[48] Gremare A, Guduric V, Bareille R, Heroguez V, Latour S, L'Heureux N, Fricain J C, Catros S and Le Nihouannen D 2017 Characterization of printed PLA scaffolds for bone tissue engineering *Journal of biomedical materials research. Part A*

[49] Kim W, Lee H, Kim Y, Choi C H, Lee D, Hwang H and Kim G 2016 Versatile design of hydrogel-based scaffolds with manipulated pore structure for hard-tissue regeneration *Biomedical materials (Bristol, England)* **11** 055002

[50] Kim W J, Yun H S and Kim G H 2017 An innovative cell-laden alpha-TCP/collagen scaffold fabricated using a two-step printing process for potential application in regenerating hard tissues *Sci Rep* **7** 3181

[51] Serra T, Planell J A and Navarro M 2013 High-resolution PLA-based composite scaffolds via 3-D printing technology *Acta biomaterialia* **9** 5521-30

[52] Buyukhatipoglu K, Chang R, Sun W and Clyne A M 2010 Bioprinted nanoparticles for tissue engineering applications *Tissue engineering. Part C, Methods* **16** 631-42

[53] Colosi C, Shin S R, Manoharan V, Massa S, Costantini M, Barbetta A, Dokmeci M R, Dentini M and Khademhosseini A 2016 Microfluidic Bioprinting of Heterogeneous 3D Tissue Constructs Using Low-Viscosity Bioink *Advanced Materials* **28** 677-84

[54] Duan B, Kapetanovic E, Hockaday L A and Butcher J T 2014 Three-dimensional printed trileaflet valve conduits using biological hydrogels and human valve interstitial cells *Acta biomaterialia* **10** 1836-46

[55] Kang H W and Lee S J 2016 A 3D bioprinting system to produce human-scale tissue constructs with structural integrity **34** 312-9

[56] Chiono V, Mozetic P, Boffito M, Sartori S, Gioffredi E, Silvestri A, Rainer A, Giannitelli S M, Trombetta M, Nurzynska D, Di Meglio F, Castaldo C, Miraglia R, Montagnani S and Ciardelli G 2014 Polyurethane-based scaffolds for myocardial tissue engineering *Interface focus* **4** 20130045

[57] Khalil S and Sun W 2009 Bioprinting endothelial cells with alginate for 3D tissue constructs *Journal of biomechanical engineering* **131** 111002

[58] You F, Wu X, Zhu N, Lei M, Eames B F and Chen X 2016 3D Printing of Porous Cell-Laden Hydrogel Constructs for Potential Applications in Cartilage Tissue Engineering *ACS Biomaterials Science & Engineering* **2** 1200-10

[59] Akkineni A R, Ahlfeld T, Lode A and Gelinsky M 2016 A versatile method for combining different biopolymers in a core/shell fashion by 3D plotting to achieve mechanically robust constructs *Biofabrication* **8** 045001

[60] Kesti M, Eberhardt C, Pagliccia G, Kenkel D, Grande D, Boss A and Zenobi-Wong M 2015 Bioprinting Complex Cartilaginous Structures with Clinically Compliant Biomaterials *Advanced Functional Materials* **25** 7406-17

[61] Akkineni A, Ahlfeld T, Funk A, Waske A, Lode A and Gelinsky M 2016 Highly Concentrated Alginate-Gellan Gum Composites for 3D Plotting of Complex Tissue Engineering Scaffolds *Polymers* **8** 170

[62] Costantini M, Idaszek J, Szoke K, Jaroszewicz J, Dentini M, Barbetta A, Brinchmann J E and Swieszkowski W 2016 3D bioprinting of BM-MSCs-loaded ECM biomimetic hydrogels for in vitro neocartilage formation *Biofabrication* **8** 035002

[63] Song B R, Yang S S, Jin H, Lee S H, Park D Y, Lee J H, Park S R, Park S-H and Min B-H 2015 Three dimensional plotted extracellular matrix scaffolds using a rapid prototyping for tissue engineering application *Tissue Engineering and Regenerative Medicine* **12** 172-80

[64] Levato R, Visser J, Planell J A, Engel E, Malda J and Mateos-Timoneda M A 2014 Biofabrication of tissue constructs by 3D bioprinting of cell-laden microcarriers *Biofabrication* **6** 035020

[65] Mouser V H, Abbadessa A, Levato R, Hennink W E, Vermonden T, Gawlitta D and Malda J 2017 Development of a thermosensitive HAMA-containing bio-ink for the fabrication of composite cartilage repair constructs *Biofabrication* **9** 015026

[66] Di Bella C, Duchi S, O'Connell C D, Blanchard R, Augustine C, Yue Z, Thompson F, Richards C, Beirne S, Onofrillo C, Bauquier S H, Ryan S D, Pivonka P, Wallace G G and Choong P F 2017 In situ handheld three-dimensional bioprinting for cartilage regeneration *Journal of tissue engineering and regenerative medicine*

[67] Boere K W, Visser J, Seyednejad H, Rahimian S, Gawlitta D, van Steenbergen M J, Dhert W J, Hennink W E, Vermonden T and Malda J 2014 Covalent attachment of a three-dimensionally printed thermoplast to a gelatin hydrogel for mechanically enhanced cartilage constructs *Acta biomaterialia* **10** 2602-11

[68] Visscher D O, Bos E J, Peeters M, Kuzmin N V, Groot M L, Helder M N and van Zuijlen P P 2016 Cartilage Tissue Engineering: Preventing Tissue Scaffold Contraction Using a 3D-Printed Polymeric Cage *Tissue engineering. Part C, Methods* **22** 573-84

[69] Muller M, Becher J, Schnabelrauch M and Zenobi-Wong M 2015 Nanostructured Pluronic hydrogels as bioinks for 3D bioprinting *Biofabrication* **7** 035006

[70] Gao Q, Liu Z, Lin Z, Qiu J, Liu Y, Liu A, Wang Y, Xiang M, Chen B, Fu J and He Y 2017 3D Bioprinting of Vessel-like Structures with Multilevel Fluidic Channels *ACS Biomaterials Science & Engineering* **3** 399-408

[71] Kosik-Kozioł A, Costantini M, Bolek T, Szöke K, Barbetta A, Brinchmann J and Święszkowski W 2017 PLA short sub-micron fiber reinforcement of 3D bioprinted alginate constructs for cartilage regeneration *Biofabrication* **9** 044105

[72] Censi R, Schuurman W, Malda J, di Dato G, Burgisser P E, Dhert W J A, van Nostrum C F, di Martino P, Vermonden T and Hennink W E 2011 A Printable Photopolymerizable Thermosensitive p(HPMAm-lactate)-PEG Hydrogel for Tissue Engineering *Advanced Functional Materials* **21** 1833-42

[73] Chang J W, Park S A, Park J K, Choi J W, Kim Y S, Shin Y S and Kim C H 2014 Tissue-engineered tracheal reconstruction using three-dimensionally printed artificial tracheal graft: preliminary report *Artificial organs* **38** E95-e105

[74] Kim B S, Jinah J, Suhun C, Ge G, Jeong-Sik K, Minjun A and Dong-Woo C 2016 Three-dimensional bioprinting of cell-laden constructs with polycaprolactone protective layers for using various thermoplastic polymers *Biofabrication* **8** 035013

[75] Di Luca A, Szlazak K, Lorenzo-Moldero I, Ghebes C A, Lepedda A, Swieszkowski W, Van Blitterswijk C and Moroni L 2016 Influencing chondrogenic differentiation of human mesenchymal stromal cells in scaffolds displaying a structural gradient in pore size *Acta biomaterialia* **36** 210-9

[76] Hung K C, Tseng C S and Hsu S H 2014 Synthesis and 3D printing of biodegradable polyurethane elastomer by a water-based process for cartilage tissue engineering applications *Advanced healthcare materials* **3** 1578-87

[77] Pescosolido L, Vermonden T, Malda J, Censi R, Dhert W J, Alhaique F, Hennink W E and Matricardi P 2011 In situ forming IPN hydrogels of calcium alginate and dextran-HEMA for biomedical applications *Acta biomaterialia* **7** 1627-33

[78] Billiet T, Gevaert E, De Schryver T, Cornelissen M and Dubruel P 2014 The 3D printing of gelatin methacrylamide cell-laden tissue-engineered constructs with high cell viability *Biomaterials* **35** 49-62

[79] Skardal A, Devarasetty M, Kang H W, Mead I, Bishop C, Shupe T, Lee S J, Jackson J, Yoo J, Soker S and Atala A 2015 A hydrogel bioink toolkit for mimicking native tissue biochemical and mechanical properties in bioprinted tissue constructs *Acta biomaterialia* **25** 24-34

[80] Chung J H Y, Naficy S, Yue Z, Kapsa R, Quigley A, Moulton S E and Wallace G G 2013 Bio-ink properties and printability for extrusion printing living cells *Biomaterials science* **1** 763-73

[81] Merceron T K, Burt M, Seol Y J, Kang H W, Lee S J, Yoo J J and Atala A 2015 A 3D bioprinted complex structure for engineering the muscle-tendon unit *Biofabrication* **7** 035003

[82] Rimington R P, Capel A J, Christie S D R and Lewis M P 2017 Biocompatible 3D printed polymers via fused deposition modelling direct C2C12 cellular phenotype in vitro *Lab on a chip* **17** 2982-93

[83] Costantini M, Testa S, Mozetic P, Barbetta A, Fuoco C, Fornetti E, Tamiro F, Bernardini S, Jaroszewicz J, Święszkowski W, Trombetta M, Castagnoli L, Seliktar D, Garstecki P, Cesareni G, Cannata S, Rainer A and Gargioli C 2017 Microfluidic-enhanced 3D bioprinting of aligned myoblast-laden hydrogels leads to functionally organized myofibers in vitro and in vivo *Biomaterials* **131** 98-110

[84] Choi Y J, Kim T G, Jeong J, Yi H G, Park J W, Hwang W and Cho D W 2016 3D Cell Printing of Functional Skeletal Muscle Constructs Using Skeletal Muscle-Derived Bioink *Advanced healthcare materials* **5** 2636-45

[85] Lee Y B, Polio S, Lee W, Dai G, Menon L, Carroll R S and Yoo S S 2010 Bio-printing of collagen and VEGF-releasing fibrin gel scaffolds for neural stem cell culture *Experimental neurology* **223** 645-52

[86] Tabriz A G, Hermida M A, Leslie N R and Shu W 2015 Three-dimensional bioprinting of complex cell laden alginate hydrogel structures *Biofabrication* **7** 045012

[87] Sayyar S, Cornock R, Murray E, Beirne S, Officer D L and Wallace G G 2014 Extrusion Printed Graphene/Polycaprolactone/Composites for Tissue Engineering *Materials Science Forum* **773-774** 496-502

[88] Ng W L, Yeong W Y and Naing M W 2016 Polyelectrolyte gelatin-chitosan hydrogel optimized for 3D bioprinting in skin tissue engineering *2016* **2** 10

[89] Yeo M, Lee J S, Chun W and Kim G H 2016 An Innovative Collagen-Based Cell-Printing Method for Obtaining Human Adipose Stem Cell-Laden Structures Consisting of Core-Sheath Structures for Tissue Engineering *Biomacromolecules* **17** 1365-75

[90] Schutz K, Placht A M, Paul B, Bruggemeier S, Gelinsky M and Lode A 2015 Three-dimensional plotting of a cell-laden alginate/methylcellulose blend: towards biofabrication of tissue engineering constructs with clinically relevant dimensions *Journal of tissue engineering and regenerative medicine*

[91] Elviri L, Foresti R, Bergonzi C, Zimetti F, Marchi C, Bianchera A, Bernini F, Silvestri M and Bettini R 2017 Highly defined 3D printed chitosan scaffolds featuring improved cell growth *Biomedical materials (Bristol, England)* **12** 045009

[92] Highley C B, Rodell C B and Burdick J A 2015 Direct 3D Printing of Shear-Thinning Hydrogels into Self-Healing Hydrogels *Advanced Materials* **27** 5075-9

[93] Ouyang L, Highley C B, Rodell C B, Sun W and Burdick J A 2016 3D Printing of Shear-Thinning Hyaluronic Acid Hydrogels with Secondary Cross-Linking *ACS Biomaterials Science & Engineering* **2** 1743-51

[94] Dubbin K, Tabet A and Heilshorn S C 2017 Quantitative criteria to benchmark new and existing bio-inks for cell compatibility *Biofabrication* **9** 044102

[95] Schuurman W, Khristov V, Pot M W, van Weeren P R, Dhert W J and Malda J 2011 Bioprinting of hybrid tissue constructs with tailorable mechanical properties *Biofabrication* **3** 021001

[96] Korpela J, Kokkari A, Korhonen H, Malin M, Narhi T and Seppala J 2013 Biodegradable and bioactive porous scaffold structures prepared using fused deposition modeling *Journal of biomedical materials research. Part B, Applied biomaterials* **101** 610-9

[97] Lin H-H, Hsieh F-Y, Tseng C-S and Hsu S-h 2016 Preparation and characterization of a biodegradable polyurethane hydrogel and the hybrid gel with soy protein for 3D cell-laden bioprinting *Journal of Materials Chemistry B* **4** 6694-705

[98] Chen Q, Mangadlao J D, Wallat J, De Leon A, Pokorski J K and Advincula R C 2017 3D Printing Biocompatible Polyurethane/Poly(lactic acid)/Graphene Oxide Nanocomposites: Anisotropic Properties *ACS Applied Materials & Interfaces* **9** 4015-23

[99] Echalier C, Levato R, Mateos-Timoneda M A, Castano O, Dejean S, Garric X, Pinese C, Noel D, Engel E, Martinez J, Mehdi A and Subra G 2017 Modular bioink for 3D printing of biocompatible hydrogels: sol-gel polymerization of hybrid peptides and polymers *RSC Advances* **7** 12231-5

[100] Yi-Cheun Y, Christopher B H, Liliang O and Jason A B 2016 3D printing of photocurable poly(glycerol sebacate) elastomers *Biofabrication* **8** 045004

[101] Markovic M, Van Hoorick J, Holzl K, Tromayer M, Gruber P, Nurnberger S, Dubruel P, Van Vlierberghe S, Liska R and Ovsianikov A 2015 Hybrid Tissue Engineering Scaffolds by Combination of Three-Dimensional Printing and Cell Photoencapsulation *Journal of nanotechnology in engineering and medicine* **6** 0210011-210017

[102] Lee H, Yoo J J, Kang H W and Cho D W 2016 Investigation of thermal degradation with extrusion-based dispensing modules for 3D bioprinting technology *Biofabrication* **8** 015011

[103] Mironov A V, Grigoryev A M, Krotova L I, Skaletsky N N, Popov V K and Sevastianov V I 2017 3D printing of PLGA scaffolds for tissue engineering *Journal of biomedical materials research. Part A* **105** 104-9

[104] Gettler B C, Zakhari J S, Gandhi P S and Williams S K 2017 Formation of Adipose Stromal Vascular Fraction Cell-Laden Spheroids Using a Three-Dimensional Bioprinter and Superhydrophobic Surfaces *Tissue engineering. Part C, Methods* **23** 516-24

[105] Guo R, Merkel A R, Sterling J A, Davidson J M and Guelcher S A 2015 Substrate modulus of 3D-printed scaffolds regulates the regenerative response in subcutaneous implants through the macrophage phenotype and Wnt signaling *Biomaterials* **73** 85-95

[106] Bhuthalingam R, Lim P Q, Irvine S A, Agrawal A, Mhaisalkar P S, An J, Chua C K and Venkatraman S 2015 A novel 3D printing method for cell alignment and differentiation *2015* **1** 9

[107] Ouyang L, Yao R, Mao S, Chen X, Na J and Sun W 2015 Three-dimensional bioprinting of embryonic stem cells directs highly uniform embryoid body formation *Biofabrication* **7** 044101

[108] Zhao Y, Li Y, Mao S, Sun W and Yao R 2015 The influence of printing parameters on cell survival rate and printability in microextrusion-based 3D cell printing technology *Biofabrication* **7** 045002

[109] Ouyang L, Yao R, Zhao Y and Sun W 2016 Effect of bioink properties on printability and cell viability for 3D bioplotting of embryonic stem cells *Biofabrication* **8** 035020

[110] Ding H, Tourlomousis F and Chang R C 2017 Bioprinting multidimensional constructs: a quantitative approach to understanding printed cell density and redistribution phenomena *Biomedical Physics & Engineering Express* **3** 035016

[111] Wu Z, Su X, Xu Y, Kong B, Sun W and Mi S 2016 Bioprinting three-dimensional cell-laden tissue constructs with controllable degradation *Scientific Reports* **6** 24474

[112] Liu W, Zhang Y S, Heinrich M A, De Ferrari F, Jang H L, Bakht S M, Alvarez M M, Yang J, Li Y-C, Trujillo-de Santiago G, Miri A K, Zhu K, Khoshakhlagh P, Prakash G, Cheng H, Guan X, Zhong Z, Ju J, Zhu G H, Jin X, Shin S R, Dokmeci M R and Khademhosseini A 2017 Rapid Continuous Multimaterial Extrusion Bioprinting *Advanced Materials* **29** 1604630-n/a

[113] Skardal A, Zhang J and Prestwich G D 2010 Bioprinting vessel-like constructs using hyaluronan hydrogels crosslinked with tetrahedral polyethylene glycol tetracrylates *Biomaterials* **31** 6173-81

[114] Rutz A L, Hyland K E, Jakus A E, Burghardt W R and Shah R N 2015 A Multimaterial Bioink Method for 3D Printing Tunable, Cell-Compatible Hydrogels *Advanced Materials* **27** 1607-14

[115] Jia W, Gungor-Ozkerim P S, Zhang Y S, Yue K, Zhu K, Liu W, Pi Q, Byambaa B, Dokmeci M R, Shin S R and Khademhosseini A 2016 Direct 3D bioprinting of perfusable vascular constructs using a blend bioink *Biomaterials* **106** 58-68

[116] Mistry P, Aied A, Alexander M, Shakesheff K, Bennett A and Yang J 2017 Bioprinting Using Mechanically Robust Core-Shell Cell-Laden Hydrogel Strands *Macromolecular bioscience* **17**

[117] Marchioli G, Luca A D, de Koning E, Engelse M, Van Blitterswijk C A, Karperien M, Van Apeldoorn A A and Moroni L 2016 Hybrid Polycaprolactone/Alginate Scaffolds Functionalized with VEGF to Promote de Novo Vessel Formation for the Transplantation of Islets of Langerhans *Advanced healthcare materials* **5** 1606-16

[118] Shanjani Y, Pan C C, Elomaa L and Yang Y 2015 A novel bioprinting method and system for forming hybrid tissue engineering constructs *Biofabrication* **7** 045008
